# Comparative psychometric evaluation of the Arabic version of four patient-reported outcome measures for sleep assessment: a construct validity study using Rasch analysis

**DOI:** 10.1007/s11325-025-03554-2

**Published:** 2025-12-22

**Authors:** Monira I. Aldhahi, Luigi Tesio, Stefano Scarano, Hadeel R. Bakhsh, Rehab Alhasani, Bodor H. Bin Sheeha, Antonio Caronni

**Affiliations:** 1 Department of Rehabilitation Sciences, College of Health and Rehabilitation Sciences, Princess Nourah bint Abdulrahman University, P.O. Box 84428, Riyadh, 11671 Saudi Arabia; 2 Department of Biomedical Sciences for Health, Università Degli Studi Di Milano, Milan, Italy; 3 Department of Neurorehabilitation Sciences, IRCCS Istituto Auxologico Italiano, Ospedale San Luca, Milan, Italy

**Keywords:** Patient-reported outcomes, PROMIS, Arabic, Sleep impairments, Rasch model, Validation

## Abstract

**Purpose:**

This study evaluated the psychometric properties of four Arabic-language patient-reported outcome measures (PROMs) for sleep assessment: the Insomnia Severity Index (ISI), Epworth Sleepiness Scale (ESS), Pittsburgh Sleep Quality Index (PSQI), and PROMIS Sleep-Related Impairment (SRI) item bank.

**Methods:**

A sample of 314 healthy Arabic-speaking adults (mean age, SD: 29.1, 12.0 years; 82.2% female) with subclinical sleep symptoms on average was recruited from healthcare and community settings in Saudi Arabia. Of these, 165 participants (52.5%) were recruited from healthcare facilities, while the remaining 149 (47.5%) were recruited from community settings via an online platform. Participants completed all sleep PROMs with sociodemographic data. Rasch analysis assessed the functioning of the rating scale, model fit, dimensionality, differential item functioning (DIF), item-person targeting, and reliability.

**Results:**

The ISI showed one misfitting item, low person reliability (0.68), and poor targeting, limiting its use for mild sleep impairment. The ESS demonstrated ordered categories and acceptable fit but reduced reliability (0.73) and poor targeting. The PSQI demonstrated acceptable item fit; however, it also showed very low reliability (0.48) and poor targeting. The original 16-item PROMIS-SRI was shortened to a 13-item version by removing three items due to poor Rasch model fit. The revised 13-item PROMIS-SRI exhibited robust psychometric properties, including well-ordered categories and thresholds, proper fit to the model, negligible multidimensionality, no substantial DIF, acceptable targeting, and high reliability (0.90).

**Conclusions:**

Among the four tested PROMs, the 13-item questionnaire from the PROMIS SRI item bank was the most psychometrically sound tool for assessing sleep-related impairment in Arabic-speaking populations. Although the ISI, ESS, and PSQI are commonly used to quantify sleep disturbance, our analysis highlighted psychometric limitations within this subclinical sample, suggesting caution in their application, particularly for detecting mild sleep disturbances.

**Supplementary Information:**

The online version contains supplementary material available at 10.1007/s11325-025-03554-2.

## Introduction

Sleep quality plays a significant role in an individual’s health and well-being. It has been reported that sleep disturbance is common in Saudi Arabia [1] and has been associated with many physical health issues, including cardiovascular diseases, metabolic disorders, and impaired immune responses [2]. Conversely, adequate sleep has been shown to enhance both physical and cognitive performance significantly [3].

Disturbances in sleep quality are attributed to a wide array of factors, including environmental settings, psychological states, and health conditions [4–7]. Sleep problems are highly prevalent across all age groups and pose a substantial global health challenge [8]. Disturbances can occur in many ways; for example, people may experience insomnia (characterized by trouble starting or maintaining sleep) or non-restorative sleep [9]. Insomnia can stem from a number of risk factors including medical and mental health conditions, lifestyle and stressful events [10–15].

Focusing on the Arab population, sleep impairment and insomnia are prevalent not only in patient populations [16] but also in non-clinical samples, such as adolescents [17, 18], university students, and healthcare workers [19].

Regarding this last point, sleep problems affect a substantial proportion of university students in various Arab nations [20, 21]. In Saudi Arabia, sleep deprivation (less than 7 h/night) was recorded in nearly half of adolescents, with more than half feeling that they experience sleep problems [17, 18]. A cross-sectional study conducted in Tunisia revealed that more than half of adults reported poor sleep quality, with insomnia and excessive daytime sleepiness as specific problems [22].

These findings highlight the importance of valid instruments for evaluating and diagnosing sleep patterns and perceived sleep quality in the future. In contemporary healthcare, patient-reported outcome measures (PROMs) offer an immediate evaluation of a patient’s perspective on their health condition, symptoms, functional capabilities, and quality of life [23]. PROMs facilitate patient-centred care by engaging patients in treatment planning and decision-making processes [24]. Integrating PROMs into clinical practice facilitates a comprehensive understanding of the multifaceted impacts of sleep disturbances and bridges the gap between subjective sleep experiences and objective clinical assessments [24].

In recent years, attention has increased on sleep-related PROMs and their psychometric properties in Arabic-speaking countries [25, 26]. Sleep-related outcome measures are invaluable for evaluating sleep disturbances, such as insomnia, excessive daytime sleepiness, and poor overall sleep quality. Several studies have examined construct validity and reliability, and have adapted existing sleep assessment tools for the Arabic-speaking population, accounting for cultural and linguistic factors [27]. The most commonly used instruments are the Insomnia Severity Index (ISI) [28], Epworth Sleepiness Scale (ESS) [26], and Pittsburgh Sleep Quality Index (PSQI) [29]. The Arabic version of the Patient-Reported Outcomes Measurement Information System (PROMIS) sleep-related impairment (SRI) item bank was also translated and culturally adapted into Arabic. However, its psychometric properties have not been tested yet [30]. These four chosen PROMs were selected because of their popularity among Arabic-speaking contexts, in addition to covering the overall sleep dimension: the ISI assesses insomnia severity, the ESS quantifies daytime sleepiness, the PSQI evaluates overall sleep quality, and the PROMIS-SRI measures sleep-related functional impairment (i.e., its effects on daily life, fatigue, and cognition). Limited research has compared the psychometric properties of different Arabic PROMs measuring the same or related variables. Such studies would be paramount in helping clinicians select an appropriate assessment tool for their specific needs.

Rasch analysis is a robust and rigorous measurement model for assessing the psychometric properties of cumulative questionnaires (“scales”) across fields, such as education, psychology, and healthcare [31]. It examines how individuals respond to assessment items to estimate their latent abilities and traits accurately [32]. Specifically, in the context of sleep-related outcome measures, Rasch analysis can provide insight into several critical psychometric properties, such as scale validity (indicated by satisfactory data-model fit), unidimensionality, item hierarchy, measure reliability, and differential item functioning (DIF, i.e., changes in some items’ difficulty across classes of respondents) [33].

Comparative validation studies are necessary to determine the relative efficacy and utility of scales that measure the same construct. The ISI, ESS, PSQI, and PROMIS SRI quantify different aspects of sleep-related impairments; however, no such comparative psychometric assessment has been conducted on the Arabic versions of these instruments. Therefore, this study aimed to assess and compare the psychometric properties of these four scales. To this aim, the questionnaires were analysed using the Rasch statistical model to provide a rigorous and robust evaluation of their measurement functioning.

## Methods

This cross-sectional methodological study evaluated the psychometric properties of four PROMs: the PROMIS SRI item banks, ISI, ESS, and PSQI. In April 2022, we obtained licenses and authorisation from the PROMIS Health Organisation to use the SRI item banks and the required permission to use the Arabic version of the other three questionnaires.

### Participants and procedure

**Fig. 1:**
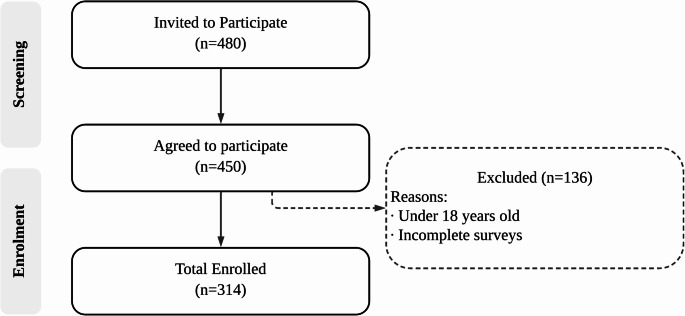
Flow diagram of participant recruitment

Participants were recruited from various settings, including outpatient clinics and community health centres through an online survey. Participants who declined to participate in the study or were unable to read Arabic were excluded. Participants with a history of severe medical conditions or psychiatric disorders were excluded based on self-reported information obtained during the initial screening. Figure 1 illustrates the participant recruitment process.

All four PROMs were administered to each participant on the same day using a fixed order, primarily for administrative convenience. The study protocol was approved by the Institutional Review Board (IRB) Ethical Committee of Princess Nourah bint Abdulrahman University (PNU 22–167) in Riyadh, Kingdom of Saudi Arabia, and all participants provided their informed consent. This study was conducted in accordance with the principles of the Declaration of Helsinki.

### Sociodemographic variables

Data on several key sociodemographic factors and lifestyle characteristics, including sex, age, educational level, and physical activity, were collected. Additionally, all participants were asked to report any health-related issues.

### Patient-reported outcome measures

All four patient-reported outcome measures (PROMs) were administered in Modern Standard Arabic (MSA), also known as Fusha. This approach was chosen to ensure standardised comprehension across diverse Arabic-speaking populations, thereby minimizing the influence of local dialect variations and potential misinterpretations of the questions.

#### Insomnia severity index (ISI)

The ISI is a self-report questionnaire designed to assess the nature and severity of sleep disturbance over the previous two weeks [28]. It consists of seven items, each scored from 0 to 4, with higher scores indicating greater severity of insomnia.The Arabic version of the ISI was used in this study [28].

#### Epworth sleepiness scale (ESS)

The ESS arabic version assesses daytime sleepiness and aids in the diagnosis of sleep disorders. It comprises eight items that assess the likelihood of dozing off or falling asleep in various daily situations [26].

#### PROMIS Sleep-Related impairments (SRI)

The PROMIS Sleep-Related Impairment item bank, part of the Patient-Reported Outcomes Measurement Information System (PROMIS), comprises 16 items that quantify the impact of sleep issues on waking hours by targeting patients’ sleepiness, fatigue, and cognitive difficulties. The SRI item bank is culturally and linguistically validated for use in Arab countries [30].

#### Pittsburgh sleep quality index (PSQI)

The PSQI, also available in Arabic [29], is a widely used instrument for assessing overall sleep quality and disturbances over a one-month period [34]. The index comprises 19 items across seven components of the study.

### Data analysis

Data cleaning, preparation, and descriptive statistics were performed using STATA version 17 (StataCorp, College Station TX, USA). Rasch analysis was conducted using the Winsteps^®^ software version 4.8.0. Graphics and additional statistics were generated using R (version 4.3.2). The four PROMs were assessed separately using Rasch analysis with an iterative approach, as customary. Specifically, upon identifying a major flaw in the questionnaire, the analysis was halted, a solution was implemented, and a new analysis was initiated. The following steps were performed:

#### Rating scale functioning

Rating scale functioning refers to the ordering of response categories. A higher category numeral should reflect an increase in the underlying variable (e.g., a participant’s ability or the severity of a condition). The same holds for the modal “thresholds,” the latent trait level at which adjacent categories are equally likely. The mean measure of the categories and the modal threshold order [35] were assessed to evaluate category functioning. Finding disordered category measures or disordered thresholds flags a major flaw in the scale’s conceptualisation.

#### Model fit

Rasch residuals are calculated as the squared differences between observed values and the values predicted by the Rasch model. The mean square (MNSQ) and z-standardised (ZSTD) outfit and infit statistics were used to quantify the magnitude of misfit between the observed data and the model’s predictions and to assess their statistical significance, respectively. Outfit (i.e., unweighted) statistics are sensitive to outliers (i.e., responses to items with difficulties that are distant from respondents, and vice versa). In contrast, as an information-weighted measure of fit, the infit statistic is primarily sensitive to unexpected response patterns on items targeted at a person’s specific ability level, i.e., where the model itself expects some random variance.

MNSQ fit statistics provide information on the size of the unexpected data. To compute an item’s MNSQ, the squared standardized residuals are calculated for all observations and averaged; MNSQ equals to the chi-square statistic divided by the degrees of freedom. Its ideal value is 1.0, with lower values indicating too predictable observations and values greater than 1.0 indicating unpredictability. A statistically acceptable fit is defined as MNSQ values ranging from 0.5 to 1.5, with the associated standardized z values (ZSTD) in the range of ± 2.0 unit-normal deviates (approximately a 0.05 significance level) [36]. The MNSQ significance changes with sample size when converted to a chi-squared test.

The rating scale model was used to analyse the PROMIS-SRI and ESS questionnaires. The grouped rating scale model was used for the ISS, as groups of items (1–3, 5–7, and item 4) shared the same category structure [35]. Finally, the partial credit model was used for the PSQI because each item had its own category structure.

#### Dimensionality analysis

Multidimensionality can be identified through principal component analysis of standardised residuals (PCAr). In the case that a questionnaire is multidimensional, i.e., if its total score is affected by two or more latent variables, the number of hidden dimensions, i.e., the number of additional variables, is given by the number of principal components with eigenvalue > 2.0.

If multidimensionality is detected, its effects on the measures extracted from the questionnaire total scores are assessed as previously described [37].

Briefly, participants are measured with three clusters of items. Cluster 1 items had a large and positive loading on the principal component, and Cluster 3 had a large and negative loading. Cluster 2 is primarily affected by the variable grasped by the Rasch model, given that it has a negligible loading on the principal component of residuals. Participants’ measures from the three clusters were compared using analysis of variance (ANOVA). Multidimensionality does not raise concerns in measurement terms if the measures from the three clusters are not significantly different.

#### Differential item functioning (DIF)

DIF is performed to assess whether items on a test function differently, that is, they change their difficulty across distinct groups within a population, despite equal levels of the underlying trait being measured. In the current study, DIF for gender, age and physical activity level was examined. Age and gender were examined as standard demographic variables for testing invariance. The physical activity level was explicitly included specifically to verify that the items function equivalently for both active and inactive individuals. For example, fatigue from exercise versus fatigue from poor sleep could yield different item responses at similar underlying impairment levels. Confirming the absence of DIF for this variable is crucial, as it ensures that these scales can be reliably used in future studies, such as those evaluating whether a change from an inactive to an active lifestyle improves sleep.

#### Reliability and targeting

To evaluate reliability, we examined the person’s separation reliability, a Rasch analysis reliability index comparable to Cronbach’s alpha. From the reliability index, the number of “strata” was calculated, that is, the number of significantly different levels of sleep impairment a person can progress through. Finding two or (even better) more strata contradict the hypothesis that persons’ measures differ only due to randomness. Floor and ceiling effects were assessed by examining the number and percentage of participants scoring the minimum and maximum total questionnaire scores, respectively. In addition, the targeting distance —the distance between the participant’s sample mean and the item’s mean— was assessed.

## Results

### Participants characteristics

Between April 2022 and February 2023, 314 healthy participants (258 females) were enrolled in the study.

Of these, 165 participants (52.5%) were recruited from healthcare facilities and 149 participants (47.5%) from community settings. The sociodemographic characteristics of the sample are shown in Table 1. The median age of the sample was 23 years (range: 18–63 years). The majority were Saudi nationals (*n* = 300, 95.5%) and primarily resided in Riyadh, the capital of Saudi Arabia. Among the participants, 127 (42.1%) reported being physically active and 175 (57.95%) were classified as physically inactive.

**Table 1:** Demographic characteristics of the sample (*N* = 314)

Variables	
Age (years)	29.14 (12.0)
Weight (Kg)	64.59 (15.8)
Height (cm)	163 (7.8)
Body Mass Index (kg/m^2^)	24.40 (5.6)
Gender, female (*n*,%)	258 (82.2)
Education	
High School	73 (23.2%)
Bachelor degree	207 (65.9%)
Postgraduate degree	34 (10.8%)
Emplyement	
Student	149 (47.5%)
Government Employee	73 (23.2%)
Private Employee	31 (9.9%)
Self-employed	10 (3.2%)
Retired	12 (3.8%)
Unemployed	39 (12.4%)
Marital status	
Married	98 (31.2%)
Single	201 (64.0%)
Divorced	14 (4.5%)
Widowed	1 (0.3%)

The right column reports the mean (SD) for data on a ratio scale and counts (%) for nominal classifications

### Rasch analysis

#### Insomnia severity index (ISI)

A grouped rating scale model was used for the analysis. Items 1, 2, and 3 had ordered categories but disordered modal (Andrich) thresholds, while items 5, 6, and 7 had ordered categories and modal thresholds. On the contrary, item 4, “*How satisfied/dissatisfied are you with your current sleep pattern?”* had ordered modal thresholds but disordered categories. In addition, the fit of the data to the Rasch model was poor for item 4 (infit MNSQ = 1.76; infit ZSTD = 8.36; outfit MNSQ = 1.90; outfit ZSTD = 9.47). Therefore, Item 4 was removed, and a new analysis was performed. After removing item 4 and completing a second run of the analysis, all remaining items exhibited ordered categories and modal thresholds and showed a satisfactory fit to the model. The ISI questionnaire was deemed to be unidimensional, as no component from the principal component analysis of the standardised residuals had a loading greater than 2.0.

The largest DIF was found for age for item 7 (0.89 logits; *p* = 0.002), and a more modest age-related DIF was found for item two (0.62 logits). No DIF was found for sex or physical activity. Person reliability was low (0.68, “extreme” participants removed), a value sufficient for distinguishing 2.27 strata. Regarding the ceiling and floor effects, only one participant achieved the maximum score, while 19 persons (i.e., 6.1% of the sample) scored the minimum. The person’s mean measure (extreme respondents excluded) was − 0.84 (SD = 0.88) logit, indicating suboptimal ISI targeting.

**Fig. 2:**
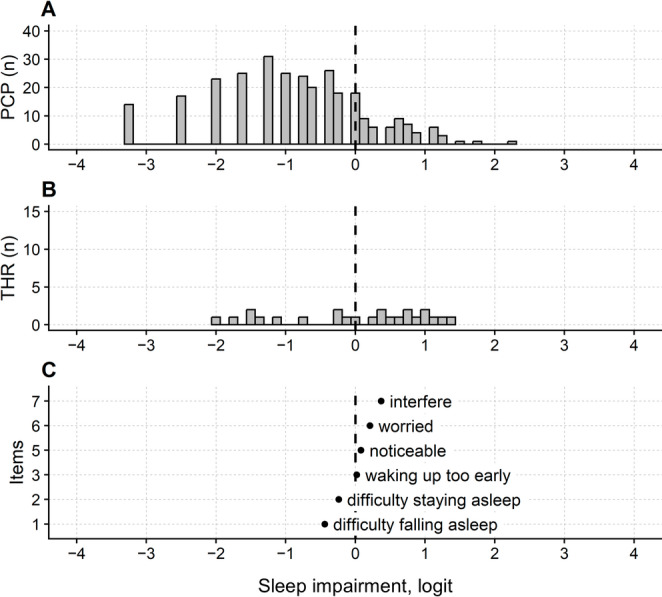
Maps of persons (**A**), thresholds (**B**), and items (**C**) of the revised Insomnia Severity Index (ISI) questionnaire. Item 4 was removed from the original ISI questionnaire because of misfit. The X-axis represents the latent construct (i.e., severity and impact of sleep disturbance), measured in logits. The severity of sleep disturbance increases from left to right. In A, persons located further to the right exhibit more severe sleep impairment. In C, items situated further to the right indicate a higher level of sleep impairment: only persons with more severe impairment are likely to endorse these items. The labels in C correspond to keywords from the ISI items. Item labels in panel C correspond, from top to bottom, to the following items: interfere (Item 7: Interference with daily functioning), worried (Item 6: Worry/distress about sleep problem), noticeable (Item 5: Noticeability of sleep problem to others), waking up too early (Item 3: Problems waking up too early), difficulty staying asleep (Item 2: Difficulty staying asleep) and difficulty falling asleep (Item 1: Difficulty falling asleep). The position of the dots in C indicates the item calibration. The vertical dashed line indicates the mean of the item calibrations, which is set to 0 logits by convention. PCP: participants; n: number of; THR: modal (Andrich) thresholds. Only non-extreme persons are reported

Figure 2 shows the persons, thresholds, and items map of the modified ISI (i.e., the six-item questionnaire after removing item 4) and highlights that there are no thresholds (i.e., no ticks on the “Rasch ruler”) for persons measuring < −2 logit (i.e., approximately 20% of the participants’ sample). These findings indicate poor precision for the ISI questionnaire in this range.

#### Epworth sleepiness scale (ESS)

All eight ESS items had ordered categories and modal thresholds (rating scale model). The fit of the data to the Rasch model was found to be satisfactory. Concerning dimensionality, the ESS looks unidimensional (principal component eigenvalues < 2.0). Item 7, *Sitting quietly after lunch*, showed a DIF for sex (contrast = 0.64 logits; *p* = 0.006). Additionally, DIF was found for age for item 4 (contrast = 0.63 logits; *p* = 0.003). No DIFs were observed for physical activity.

**Fig. 3:**
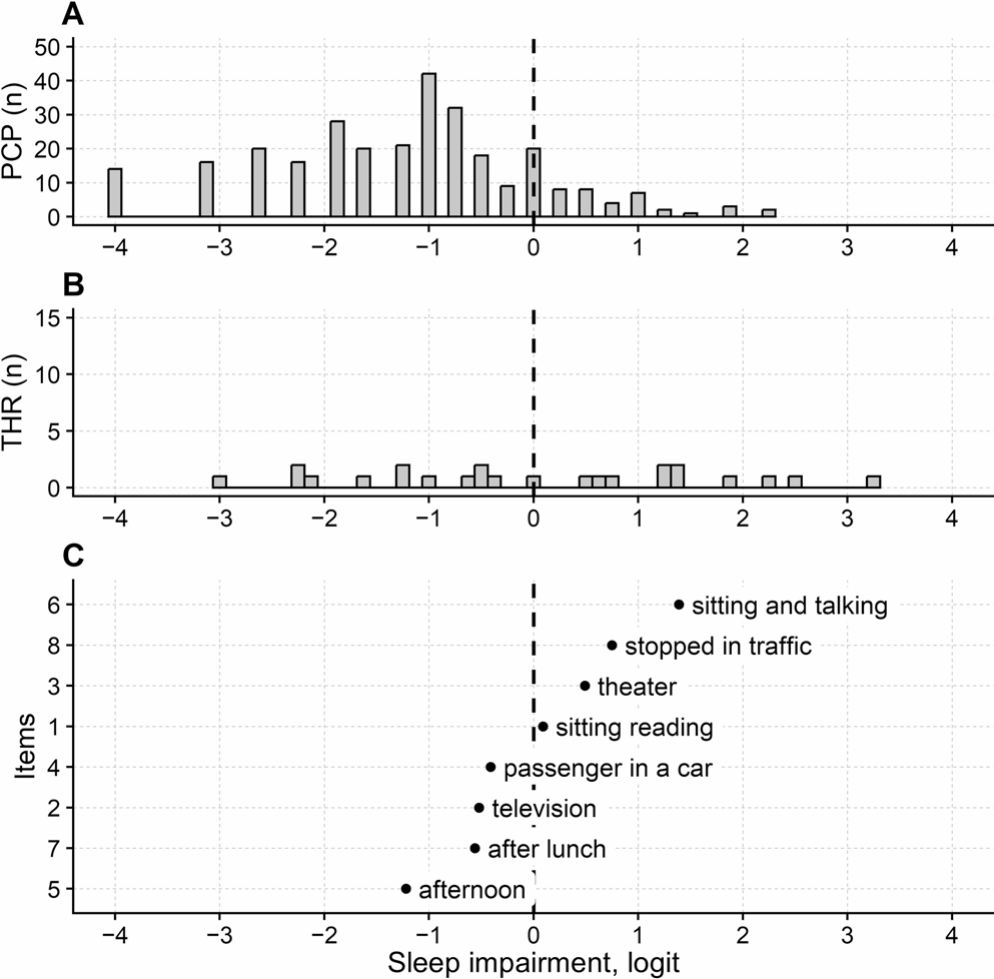
Maps of persons (**A**), thresholds (**B**), and items (**C**) of the Epworth Sleepiness Scale (ESS) questionnaire. See Fig. 2 for abbreviations and conventions. In C, item labels correspond, from top to bottom, to the following item descriptions (likelihood of dozing off): sitting and talking (Item 6: While sitting and talking to someone), stopped in traffic (Item 8: In a car, while stopped for a few minutes in traffic), theater (Item 3: Sitting in a public place, e.g., a theater or a meeting), sitting reading (Item 4: Sitting and reading), passenger in a car (Item 2: As a passenger in a car for an hour), television (Item 1: Sitting and watching television), after lunch (Item 7: Sitting quietly after lunch) and afternoon (Item 5: Lying down to rest in the afternoon)

Person reliability was low (0.73; 2.53 strata), and the questionnaire suffered some floor effect, with 22 out of 314 participants scoring the minimum score. The mean (SD) person’s measure was − 1.22 (1.26) logit (non-extreme participants only); hence, the questionnaire showed poor targeting. Figure 3 shows the persons, thresholds, and items map of the ESS.

#### PROMIS Sleep-Related impairment (PROMIS-SRI)

An initial analysis encompassing all 16 items (rating scale model) was conducted, revealing that the PROMIS-SRI items exhibited ordered categories and modal thresholds. However, items 13—Sleep119 *(“I felt alert when I woke up”)*, 14—Sleep120 *(“When I woke up I felt ready to start the day”)*, and 1—Sleep4 *(“I had enough energy”)* seriously misfitted the model (infit MNSQ > 2.1; infit ZSTD > 4.0). Therefore, these items were excluded from the questionnaire. Upon rerunning the analysis, the 13-item revised questionnaire demonstrated ordered categories and thresholds, with all items fitting the Rasch model.

Regarding dimensionality, the PCA calculated on the model’s residuals showed two components whose eigenvalues were > 2.0 (i.e., 2.54 for the first and 2.03 for the second one), thus suggesting that the questionnaire’s score is at least tri-dimensional, i.e., it depends on two more dimensions beyond the one grasped by the Rasch model.

**Fig. 4:**
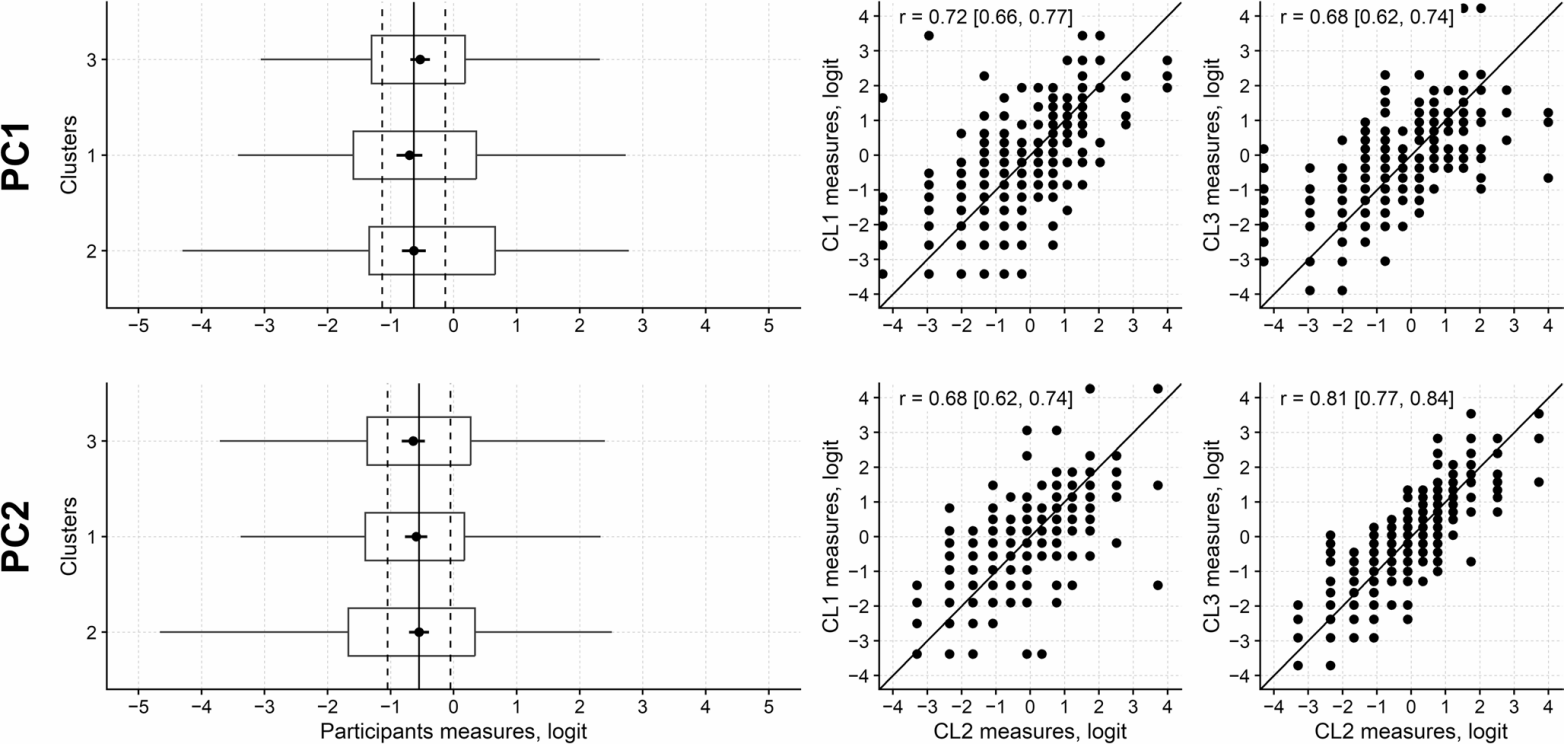
Multidimensionality of the revised 13-item PROMIS SRI and its effect on measurements. Left plots (**A**) show the mean (black dot) and 95%CI (horizontal bars) of the participants’ measures from the first, second and third clusters of items. Vertical continuous line: cluster 2 mean measure; vertical dashed lines: cluster 2 mean measure plus and minus 0.5 logit. The box and whiskers plot shows the distribution of the sample measures. The scatter plots on the right (**B**) show the relationship between measures from cluster 2 items and clusters 1 and 3. The diagonal continuous line is the identity line. PC1: first principal component; PC2: second principal component; CL1: cluster 1; CL2: cluster 2; CL3: cluster 3. r: Pearson correlation coefficient, with its 95%CI reported between squared brackets. No substantial difference is apparent between the measures from the three clusters for both components. The correlation of clusters 1 and 3 measures and cluster 2 measures is reasonable. No substantial offset from the identity line is apparent

However, regarding the first principal component, ANOVA did not find a significant difference across the measures from the three clusters of items (F_2,616_ = 2.51; *p* = 0.083), indicating that when participants are measured with those items whose score is considerably affected by the first hidden, additional variable (i.e. Cluster 1 and Cluster 3 items), their measures are not substantially different from those returned by the items’ cluster only reflecting the variable of the Rasch model (i.e. Cluster 2 items). Comparable results were obtained for the second principal component (F_2,616_ = 0.92; *p* = 0.399; Fig. 4). Therefore, despite being present in strictly mathematical terms, the multidimensionality flagged by the first or second component does not represent severe harm to the measurement.

**Fig. 5:**
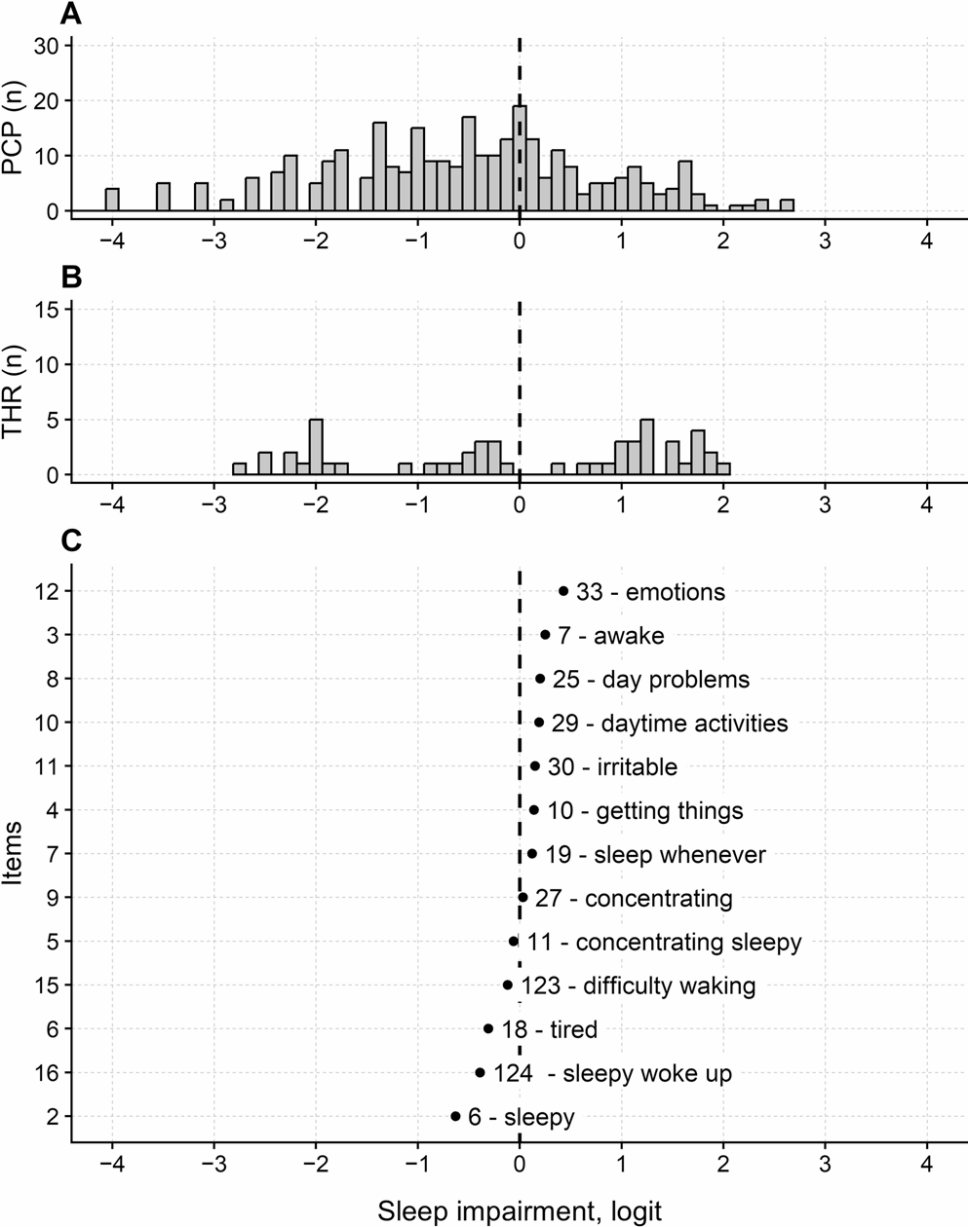
Maps of persons (**A**), thresholds (**B**), and items (**C**) of the PROMIS Sleep-Related Impairment (PROMIS^®^ SRI) questionnaire – revised. The revised PROMIS SRI was created by removing three misfitting items (1, 13, and 14) from the original version. See Fig. 2 for abbreviations and conventions. In C, item labels correspond, from top to bottom, to the following item descriptions: 33 - emotions (Item Sleep33: Interference with emotions due to sleepiness), 7 - awake (Item Sleep7: Trouble staying awake during the day), 25 - day problems (Item Sleep25: Sleepiness causing problems during the day), 29 - daytime activities (Item Sleep29: Trouble with daytime activities due to sleepiness), 30 - irritable (Item Sleep30: Irritability due to sleepiness), 10 - getting things (Item Sleep10: Difficulty getting things done due to sleepiness), 19 - sleep whenever (Item Sleep19: Dozing off or napping when given the chance), 27 - concentrating (Item Sleep27: Difficulty concentrating due to sleepiness), 11 - concentrating sleepy (Item Sleep11: Difficulty paying attention due to sleepiness), 123 - difficulty waking (Item Sleep123: Difficulty waking up), 18 - tired (Item Sleep18: Feeling tired), 124 - sleepy woke up (Item Sleep124: Feeling sleepy upon waking) and 6 - sleepy (Item Sleep6: Feeling sleepy)

The reliability of the PROMIS-SRI was good (0.9; non-extreme respondents only), allowing for the distinction of 4.3 strata. Figure 5 shows the person, threshold, and item maps of the PROMIS SRI.

Six participants (1.9% of the total sample) scored the minimum total questionnaire score. If only non-extreme individuals were considered, the mean (SD) measure was − 0.56 (1.34) logit. The score-to-measure conversion table for the revised PROMIS Sleep-Related Impairment (SRI) scale is presented in Supplementary Material, Table 1.

#### Pittsburgh sleep quality index (PSQI)

All seven components (i.e., items) of the PSQI had ordered categories, but components 3, 4, and 6 had disordered modal thresholds (partial credit models). Despite threshold disordering, the fit to the Rasch model was satisfactory for all seven components of the scale. The PSQI appeared to be unidimensional, as the PCA conducted on the model’s residuals revealed no component with an eigenvalue greater than 2.0. Component 3 (“Sleep duration”) was affected by DIF for both gender and age. No DIF was found for physical activity either.

Person Reliability was low (0.48) when only non-extreme participants were considered. With such a low reliability value, the PSQI has only one stratum (which indicates that the measures do not discriminate across individuals).

**Fig. 6:**
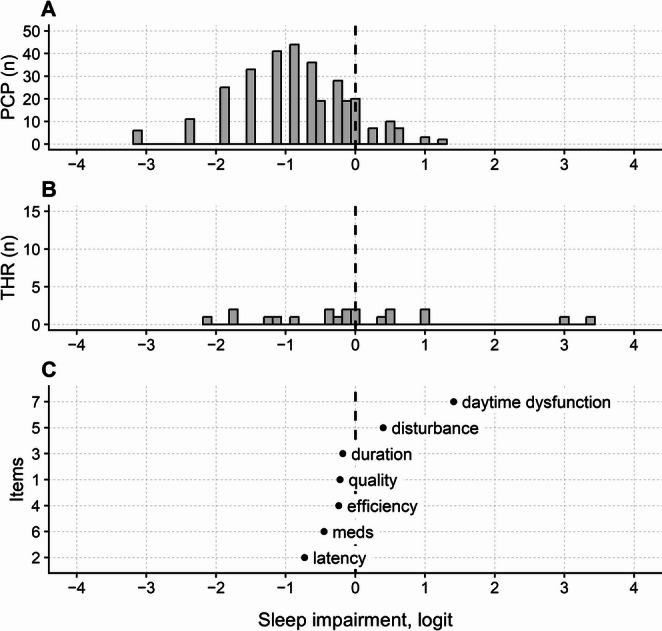
Maps of persons (**A**), thresholds (**B**), and items (**C**) of the Pittsburgh Sleep Quality Index (PSQI) questionnaire. See Fig. 2 for abbreviations and conventions. In C, item labels correspond, from top to bottom, to the following PSQI component descriptions: daytime dysfunction (Component 7: Daytime dysfunction), disturbance (Component 5: Sleep disturbance), duration (Component 3: Sleep duration), quality (Component 1: Subjective sleep quality), efficiency (Component 4: Habitual sleep efficiency), meds (Component 6: Use of sleep medication) and latency (Component 2: Sleep latency)

The PSQI persons, thresholds, and items map are shown in Fig. 6; most participants scored below zero logits, indicating that the questionnaire was not well-targeted to the sample of participants.

If only non-extreme participants were considered, the person’s mean (SD) measure was − 0.80 (0.79) logits. The threshold map (Fig. 6B) revealed a substantial gap between 1 and 3 logits, indicating a range of constructs that were poorly probed by the questionnaire’s components.

A summary of the Rasch analysis findings for all four sleep-related outcome measures, including item fit, response category functioning, dimensionality, DIF, and person reliability, is presented in Table 2. All numerical details, including thresholds, item calibrations, item fit, and results of the dimensionality and DIF analyses, are reported in the Supplementary Materials.

**Table 2:** Summary of Rasch analysis results for the four Sleep-Related outcome measures

Questionnaire	Categories’ mean measure	Modal thresholds	Fit to the model	Dimensionality	DIF	Items-persons targeting (SD)	Persons measures reliability (strata)
ISI revised (6 items)	Ordered	Ordered	Proper	Unidimensional	Age (item 7, 0.89 logits; item 2, 0.62 logits)	−0.84 (1.07)	0.68 (2.27)
ESS	Ordered	Ordered	Proper	Unidimensional	Gender (item 7, 0.64 logit) Age (item 4, 0.63 logits)	−1.22 (1.26)	0.73 (2.51)
PROMIS-SRI original	Ordered	Ordered	Three items misfitting (4, 118 and 120)	-	-	-	-
PROMIS-SRI revised (13 items)	Ordered	Ordered	Proper	Slight multidimensionality	None	−0.56 (1.34)	0.90 (4.37)
PSQI	Ordered	Disordered for components 3,4,6	Proper	Unidimensional	Gender (component 3, 0.55 logits) Age (component 3, 0.90 logits)	−0.80 (0.79)	0.48 (1.61)

ISI: Insomnia Severity Index; ESS: Epworth Sleepiness Scale; PROMIS−SRI: PROMIS Sleep−Related Impairment questionnaire; PSQI: Pittsburgh Sleep Quality Index; DIF: Differential Item Functioning. The PROMIS−SRI and ESS questionnaires were analysed using the rating scale model, while the grouped rating scale model was employed for the ISS. The partial credit model was used for the PSQI. Dimensionality, DIF, targeting, and reliability were not assessed for the original PROMIS−SRI, as the analysis was stopped due to misfitting items. DIF was tested for gender, age, and physical activity. Targeting and reliability are calculated considering only non−extreme participants. Specifically, for the revised 13−item PROMIS−SRI, 6 persons (1.9%) achieved the minimum score. For the ISI, 1 person (0.3%) achieved the maximum score and 19 (6.1%) the minimum. For the ESS, 1 person (0.3%) achieved the maximum score and 22 (7.0%) the minimum. For the PSQI, 3 persons (1.0%) achieved the minimum score. The slight multidimensionality found in the 13−item revised PROMIS−SRI questionnaire poses no concerns in measurement terms

## Discussion

The Rasch analysis revealed clear differences in measurement performance across the four Arabic-language questionnaires. The revised 13-item PROMIS-SRI emerged as the most psychometrically sound instrument, demonstrating robust properties, including ordered categories, good model fit, and high person reliability. In contrast, the other instruments showed significant limitations in their psychometric properties. Specifically, the original ISI exhibited item misfit, and the PSQI had disordered response categories. Furthermore, the ISI, ESS, and PSQI were all constrained by low reliability and poor person-item targeting, limiting their accuracy in this sample with mild or subclinical sleep issues.

However, reliability and targeting depend not only on the questionnaire but also on the recruited sample. It is sufficient to recall that the reliability of person measures is the ratio of person variance to the total variance of their measures, while targeting is the difference between the mean person ability measure and the mean item difficulty. Therefore, to achieve better reliability and targeting in this study, it would have been sufficient to recruit a sample with more severe sleep disturbances, thus increasing the variance in severity levels. Notably, our sample consisted mainly of young women with no known medical conditions who were unlikely to have a clinically relevant sleep disorder. However, participants who scored the minimum, that is, those without any sleep disturbance according to the questionnaires, were automatically excluded from item calibration, which constitutes the core of Rasch analysis. Moreover, they were deliberately excluded from the reliability and targeting calculations. Thus, we can state that all participants included in the analysis reported some degree of sleep disturbance.

The study of even mild sleep disturbances is justified from a clinical standpoint; therefore, assessing the performance of these questionnaires in a subclinical population is also recommended. First, a non-negligible proportion of people experience mild sleep complaints, which can negatively impact their daily lives [38]. Consequently, it is unsurprising that clinical trials have evaluated treatments to improve sleep disturbances, even in individuals with minimal symptoms [39], highlighting the importance of having sound measures of sleep in this subpopulation. Second, the questionnaires assessed here are often used as screening tools [40, 41], suggesting that they are also intended for patients who may not have a sleep disorder. When used as a screening tool, it is noteworthy in the current context that the cutoff for distinguishing impaired individuals from controls is not the minimum total score, at least for some questionnaires [40]. This finding further emphasises the importance of the questionnaires functioning accurately, even at the extremes of a clinical condition. Moreover, the evaluation of these questionnaires in samples of patients with mild sleep impairment is relatively common, and prior studies have recruited healthy controls, including very young individuals, for this specific aim. For example, PROMIS-SRI has been suggested as a valid measure of sleep impairment, not only in patients known to suffer from sleep impairment, such as those with Parkinson’s disease, but also in middle-aged controls [42]. In addition, PROMIS sleep questionnaires have been validated in young and healthy individuals, such as adolescents recruited from secondary schools [43], and are considered to properly distinguish between clinical and nonclinical groups of young people [44].

Regarding minimal sleep disturbances, it is appropriate to note that many substantially healthy individuals, such as those recruited in this study, may suffer from such disturbances due to poor sleep hygiene. Contemporary factors, such as blue light emitted by screens, such as smartphones and tablets, can alter the sleep cycle. The ubiquity and intensive use of these devices make it even more important to validate psychometric instruments for assessing sleep in this subclinical population [4].

This discussion on sample-dependent statistics, such as reliability and targeting, leads to crucial methodological reflections. A central claim of Rasch analysis is that its measures and calibrations are independent of the specific sample recruited and the specific items used—a property known as “specific objectivity.” Therefore, it is tempting to conclude that the characteristics of the recruited sample are irrelevant to the validation process.

However, we argue that this is a dangerous oversimplification. The theoretical sample-free properties of Rasch measures hold only in the “mathematically pure” case of perfect data-to-model fit. This is an abstract ideal that is invariably violated by real-world data. Therefore, the goal of the analysis must be to empirically test this assumed invariance rather than taking it for granted. The entire framework of DIF testing, for instance, is a direct empirical test of this invariance property across different person subgroups.

In other words, the theory dictates that if the model holds, the measures are sample-independent. Since perfect model-data fit is never guaranteed, the analyst’s task is to empirically verify this invariance rather than to assume it a priori.

Overall, the revised PROMIS-SRI demonstrated ordered category average measures and ordered modal thresholds, with items fitting the Rasch model properly. It was affected only by slight multidimensionality, which had no impact on the measures derived from the total score of the questionnaire. Its items showed no DIF for essential variables, such as age and sex. In stark contrast to other sleep questionnaires, the revised PROMIS-SRI demonstrated excellent reliability (0.90), making it a measurement instrument with sufficient precision for assessing individuals.

To our knowledge, this study is the first to apply Rasch analysis to the Arabic versions of the ISI, ESS, PROMIS SRI, and PSQI questionnaires. However, Rasch analysis has been applied to other translations of these questionnaires in the past. Although our review of the literature was not systematic, our findings are largely consistent with those of previous studies. For example, the Rasch analysis of the ISI in an Iranian sample of participants showed that its items had proper infit and outfit and a person reliability index of 0.78 [2]. The Portuguese version of the ESS [45] exhibited ordered categories and modal thresholds, with a person reliability of 0.78, comparable to that found in this study. Rasch analysis of the Taiwanese PSQI [46] also showed that the questionnaire has ordered categories and well-fitting items. In contrast to our findings, the person’s reliability was higher (0.75).

The PROMIS questionnaire is a special case compared to the ISI, ESS, and PSQI, as it was initially developed using the item response theory. Consequently, it may seem inappropriate to evaluate its properties using a different statistical model, such as Rasch analysis. However, Rasch analysis of some PROMIS questionnaires has already been proposed by our [47] and other research groups [48]. More importantly, strictly speaking, Rasch models are a special case within the more general framework of item response theory.

## Study strengths and limitations

A notable strength of the current study is the first comprehensive application of Rasch model analysis to these four Arabic-language sleep outcome measures, uncovering significant variability in their psychometric properties. The use of a large, community-based Arabic-speaking sample and a rigorous Rasch analytical framework enhanced the validity of these results. Our findings identified the revised 13-item PROMIS-SRI as the most robust tool, showing proper item fit, ordered response categories, high reliability, and minimal multidimensionality.

However, this study has limitations, and the findings warrant a detailed discussion. A key finding that warrants discussion is the removal of three items from the original PROMIS-SRI to create a revised 13-item version. It is important to emphasize that while this step was necessary due to the items’ severe psychometric misfit, removing items from an established scale has potential consequences. Specifically, it risks compromising content validity by discarding questions that investigate unique facets of the construct, meaning that some aspects of sleep disturbance may become under-investigated even as the scale’s psychometric fit improves. However, the removal was strongly justified by the items’ very high infit MNSQ values (exceeding 2.0). At such levels of misfit, these items not only fail to contribute to the measurement but also actively begin to degrade or distort the measures derived from the remaining items [36]. A second implication is that using a modified questionnaire complicates comparisons with studies that have used the original instrument. This concern, however, is substantially mitigated by the nature of PROMIS itself. While we have referred to the PROMIS-SRI as a “questionnaire,” its items are technically part of an item bank. Therefore, it is inherent to the PROMIS framework that its items can be selected and assembled into various short forms or parallel versions, as was done in this study to optimize measurement.

Looking at the broader literature on PROMIS instruments, this is not the only study to find misfitting items when applying the Rasch analysis. Indeed, compared to other studies where most items were reported as misfitting [49, 50] in some cases leading to the removal of the entire item bank, the PROMIS-SRI performed remarkably well in this study. It is also worth noting that this discrepancy (i.e., the PROMIS-SRI having only three misfitting items versus other PROMIS scales with widespread misfit) may be attributable not only to the specific items but also to differences in the analytical process. In this respect, even within accepted practices, analytical decisions can be applied in more or less liberal ways.

While we already acknowledged that our sample consisted primarily of healthy females from a non-clinical setting with a narrow age range, this characteristic warrants further discussion. Validating these scales in a sample with subclinical complaints is not a methodological flaw per se. On the contrary, these instruments are frequently used precisely to screen for and quantify even minimal sleep disturbances. In this specific context, the revised ISI, ESS, and PSQI performed substantially worse than the 13-item revised PROMIS-SRI. This comparison is a central finding of our study: in this population, the revised PROMIS-SRI was the most robust of the four instruments. This study cannot conclude that the ISI, ESS, and PSQI are invalid instruments; they may function correctly in populations with clinically confirmed sleep disorders. Our findings are limited to their performance in subclinical, non-clinical samples.

In addition, all participants were recruited exclusively from a single major urban center. Therefore, the findings may not be generalisable to the broader Saudi population or Arabic-speaking individuals.

Another limitation of this study is that the four questionnaires were administered in a fixed order for practical reasons. We must acknowledge that this may have introduced a systematic bias from either a fatigue effect or a priming effect. For instance, the first questionnaire administered may have unconsciously framed how participants perceived their “sleep problem,” potentially influencing their responses to the subsequent scales.

Finally, the cross-sectional nature of the present study restricts the testing of test–retest reliability and responsiveness, which could be explored more adequately in future longitudinal studies.

Regarding the future development of the current line of research, our study indicates that refinement efforts should focus on enhancing the measurement precision of the instruments tested here, particularly at the lower end of the impairment continuum. In addition, future studies should aim to replicate this study with Arabic-speaking clinical populations diagnosed with specific sleep disorders (e.g., clinically defined insomnia and sleep apnea), which is crucial for determining the validity and reliability of the questionnaires in the context of sleep pathology. This further investigation will help establish their utility in the clinical assessment and monitoring of treatment outcomes.

## Conclusions

This study provides a psychometric evaluation of four commonly used sleep assessment PROMs in a sample of Arabic-speaking adults in Saudi Arabia using the Rasch analysis. The findings revealed varying psychometric properties across the measures, with the revised 13-item PROMIS-SRI emerging as the most psychometrically sound tool for assessing sleep-related impairment in this context.

Overall, this study provides valuable evidence supporting the use of the revised PROMIS-SRI as a reliable Arabic-language tool for measuring sleep-related impairment. Although the ISI, ESS, and PSQI are widely used, our analysis highlighted significant limitations in their reliability and targeting within this subclinical sample, suggesting caution in their application for detecting mild sleep disturbance.

Future research should prioritize the validation of these instruments in diverse clinical contexts, including patients with an overt sleep impairment, focusing on exploring their longitudinal stability (e.g. test-retest reliability) and responsiveness.

## Supplementary Information

Below is the link to the electronic supplementary material.

Supplementary file 1 (DOCX 84.0 KB)

## Data Availability

The data presented in this study are available upon request from the corresponding author. The data are not publicly available for further publication because of restrictions related to the ongoing work.

## Declarations

### Clinical trial registration

Clinical trial number is not applicable.

## Abbreviations

ESS: Epworth Sleepiness ScaleEpworth Sleepiness Scale
ISI: Insomnia Severity Index
PROs: Patient-Reported Outcome Measures
PROMIS: Patient-Reported Outcomes Measurement Information
PSQI: Pittsburgh Sleep Quality Index
SRI: Sleep related impairments
