# Supplementary material for: Comparative psychometric evaluation of the Arabic version of four patient-reported outcome measures for sleep assessment: a construct validity study using Rasch analysis
Source: Sleep Breath. 2025 Dec 22;30(1):14. doi: 10.1007/s11325-025-03554-2 (PMC12722280; doi:10.1007/s11325-025-03554-2)
Supplement: Supplementary file 1 — (DOCX 84.0 KB) [file 11325_2025_3554_MOESM1_ESM.docx]

**Comparative psychometric Evaluation of the Arabic Version of four Patient-Reported Outcome Measures for Sleep Assessment: a construct validity study using Rasch analysis**

**Supplementary Materials 1**

These supplementary materials present the detailed numerical results from the Rasch analysis of the Insomnia Severity Index (ISI), Epworth Sleepiness Scale (ESS), PROMIS Sleep-Related Impairment (PROMIS-SRI), and Pittsburgh Sleep Quality Index (PSQI) questionnaires, primarily in tabular form. These results complement and provide further detail on the findings provided in the main text.

1. ***Insomnia Severity Index***
2. *Rating scale functioning – run 1*

|  | **Score** | **Observed**  **count** | **%** | **Observed**  **average** | **Modal**  **thresholds** |
| --- | --- | --- | --- | --- | --- |
| Items 1-3 | 0 | 333 | 35 | -1.35 | - |
|  | 1 | 316 | 34 | -0.86 | -1.10 |
|  | 2 | 161 | 17 | -0.31 | 0.07 |
|  | 3 | 75 | 8 | 0.03 | 0.56 |
|  | 4 | 57 | 6 | 0.42 | ***0.48 **** |
| Item 4 | 0 | 45 | 14 | -0.73 | - |
|  | 1 | 67 | 21 | ***-0.94 **** | -1.29 |
|  | 2 | 96 | 31 | -0.67 | -0.53 |
|  | 3 | 69 | 22 | -0.42 | 0.56 |
|  | 4 | 37 | 12 | -0.24 | 1.26 |
| Items 5-7 | 0 | 294 | 31 | -1.44 | - |
|  | 1 | 290 | 31 | -0.79 | -1.15 |
|  | 2 | 210 | 22 | -0.34 | -0.27 |
|  | 3 | 94 | 10 | 0.16 | 0.62 |
|  | 4 | 54 | 6 | 0.51 | 0.80 |

**Score:** category's score. **Observed count:** the total number of times a category was endorsed.. **Observed average:** the mean of (b–d) calculated for each category, where b is the respondent's ability and d is the item difficulty, in logit units. Note that each collected score originates from the encounter of a person endowed with a particular ability b and an item with a specific difficulty d. The observed average is expected to increase with category value. To accurately evaluate category function, the effect of item difficulty must be removed. Subtracting d from b achieves this, isolating the behaviour of the response categories themselves. **Modal thresholds:** Andrich's thresholds, a latent variable level for which two adjacent categories are equally likely. Disordered categories and thresholds are shown in bold and italics and marked with an asterisk (*). The Grouped Rating Scale Model was applied to the ISI, with items 1-3 and 5-7 sharing a common rating scale, and item 4 having its unique category structure.

1. *Fit to the model – run 1*

|  |  |  | **Infit** | | **Outfit** | |
| --- | --- | --- | --- | --- | --- | --- |
| **Items** | **Calibration** | **SE** | **MNSQ** | **ZSTD** | **MNSQ** | **ZSTD** |
| 4 - current sleep pattern | -0.65 | 0.06 | ***1.76 **** | 8.36 | ***1.90 **** | 9.47 |
| 3 - waking up too early | -0.10 | 0.06 | 1.02 | 0.29 | 1.01 | 0.13 |
| 2 - difficulty staying asleep | 0.41 | 0.07 | 0.91 | -0.98 | 0.87 | -1.31 |
| 5 - noticeable | 0.18 | 0.06 | 0.87 | -1.62 | 0.87 | -1.58 |
| 1 - difficulty falling asleep | 0.12 | 0.06 | 0.84 | -1.93 | 0.83 | -1.94 |
| 7 - interfere | -0.25 | 0.06 | 0.84 | -2.13 | 0.82 | -2.39 |
| 6 - worried | 0.29 | 0.07 | 0.78 | -2.92 | 0.73 | -3.37 |

**Items:** items are summarised by keywords. **Calibration:** item's calibration. **SE:** standard error. **MNSQ:** mean square fit statistics. **ZSTD:** z-standardised fit statistics. Misfitting parameters (MNSQ ≥ 1.5 with ZSTD > 1.96) are shown in bold and italics and marked with an asterisk (*).

The analysis was repeated after removing item 4 due to the presence of disordered categories and a misfit.

1. *Rating scale functioning – run 2*

|  | **Score** | **Observed**  **count** | **%** | **Observed**  **average** | **Modal**  **thresholds** |
| --- | --- | --- | --- | --- | --- |
| Items 1-3 | 0 | 333 | 35 | -1.65 | - |
|  | 1 | 316 | 34 | -1.02 | -1.46 |
|  | 2 | 161 | 17 | -.32 | 0.03 |
|  | 3 | 75 | 8 | 0.15 | 0.67 |
|  | 4 | 57 | 6 | 0.59 | 0.76 |
| Items 5-7 | 0 | 294 | 31 | -1.79 | - |
|  | 1 | 290 | 31 | -0.95 | -1.53 |
|  | 2 | 210 | 22 | -0.33 | -0.32 |
|  | 3 | 94 | 10 | 0.35 | 0.74 |
|  | 4 | 54 | 6 | 0.70 | 1.11 |

Same abbreviations and conventions as before. Note that removing item 4, i.e. the item with disordered categories and misfitting, solved the threshold disordering of items 1, 2 and 3.

1. *Fit to the model – run 2*

|  |  |  | **Infit** | | **Outfit** | |
| --- | --- | --- | --- | --- | --- | --- |
| **Items** | **Calibration** | **SE** | **MNSQ** | **ZSTD** | **MNSQ** | **ZSTD** |
| 3 - waking up too early | -0.24 | 0.07 | 1.27 | 3.02 | 1.28 | 3.01 |
| 2 - difficulty staying asleep | 0.37 | 0.07 | 1.02 | 0.28 | 0.98 | -0.18 |
| 5 - noticeable | 0.08 | 0.07 | 0.99 | -0.07 | 0.99 | -0.04 |
| 1 - difficulty falling asleep | 0.02 | 0.07 | 0.95 | -0.51 | 0.94 | -0.60 |
| 7 - interfere | -0.44 | 0.07 | 0.92 | -1.00 | 0.91 | -1.11 |
| 6 - worried | 0.21 | 0.07 | 0.86 | -1.78 | 0.84 | -1.88 |

Same abbreviations and conventions as before. Item 3 showed a statistically significant departure from the model's prediction, as indicated by its high Infit and Outfit ZSTD values. However, the magnitude of this misfit was negligible, as its Infit and Outfit MNSQ values were within the acceptable range.

1. *Dimensionality – run 2*

The eigenvalue of the first principal component is 1.82, i.e. < 2.0, thus indicating that the Insomnia Severity Index is substantially unidimensional.

1. *Differential Item Functioning – run 2*

No Differential Item Functioning (DIF) was found for sex (males vs females) and physical activity (yes vs no).

DIF was found to be related to the participants' age. For this analysis, participants have been divided into three groups: young (≤ 30 years), middle-aged (between 30 and 50 years), and old (> 50 years). However, while DIF is present, its practical impact is likely minimal, as recent statistical simulations have demonstrated that DIF of this magnitude does not significantly distort the measurements [1].

| **Item** | **Group 1** | | **Group 2** | | **Contrast** | **p-value** |
| --- | --- | --- | --- | --- | --- | --- |
| 1 - difficulty falling asleep | Young | 0.14 | Old | -0.52 | 0.66 | 0.010 |
| 2 - difficulty staying asleep | Young | 0.63 | Middle | -0.03 | 0.65 | < 0.001 |
|  | Young | 0.63 | Old | -0.16 | 0.79 | 0.003 |
| 7 - interfere | Young | -0.59 | Old | 0.30 | 0.89 | 0.002 |

**Item:** the item affected by DIF. **Group 1** and **Group 2:** the two groups that are contrasted in the DIF analysis; the item calibration (logit) is reported for each of the two groups. **Contrast:** the difference (absolute value), expressed in logit, in the item's calibration between the two groups. **P-value:** the significance level of the DIF analysis. Item 7 - Interference exhibited the most considerable DIF effect. Items 1 and 2 were also flagged for DIF, and this effect was confirmed as genuine because it persisted even after Item 7 was removed and the analysis was repeated (not shown).

1. ***Epworth Sleepiness Scale***
2. *Rating scale functioning*

| **Score** | **Observed**  **count** | **%** | **Observed**  **average** | **Modal**  **thresholds** |
| --- | --- | --- | --- | --- |
| 0 | 1004 | 40 | -2.38 | - |
| 1 | 870 | 35 | -1.07 | -1.75 |
| 2 | 503 | 20 | -0.14 | -0.04 |
| 3 | 135 | 5 | 1.02 | 1.80 |

Same abbreviations and conventions as before. Categories and thresholds were ordered.

1. *Fit to the model*

|  |  |  | **Infit** | | **Outfit** | |
| --- | --- | --- | --- | --- | --- | --- |
| **Items** | **Calibration** | **SE** | **MNSQ** | **ZSTD** | **MNSQ** | **ZSTD** |
| 8 - stopped in traffic | 0.75 | 0.10 | 1.21 | 2.30 | 1.24 | 2.17 |
| 6 – sitting and talking | 1.39 | 0.11 | 1.17 | 1.79 | 1.10 | 0.72 |
| 4 passenger in a car | -0.41 | 0.09 | 1.04 | 0.52 | 1.04 | 0.52 |
| 3 - theatre | 0.49 | 0.09 | 0.98 | -0.20 | 1.02 | 0.27 |
| 2 - television | -0.52 | 0.09 | 0.96 | -0.54 | 1.01 | 0.09 |
| 5 - afternoon | -1.22 | 0.09 | 0.95 | -0.60 | 0.95 | -0.66 |
| 1 - sitting reading | 0.09 | 0.09 | 0.89 | -1.43 | 0.88 | -1.40 |
| 7 - after lunch | -0.56 | 0.09 | 0.88 | -1.60 | 0.88 | -1.58 |

Same abbreviations and conventions as above.

1. *Dimensionality*

The eigenvalue of the first principal component is 1.78, thus indicating unidimensionality.

1. *Differential Item Functioning*

| **Item** | **Group 1** | | **Group 2** | | **Contrast** | **p-value** |
| --- | --- | --- | --- | --- | --- | --- |
| 7 - after lunch | Males | -1.09 | Females | -0.44 | 0.64 | 0.006 |
| 4 passenger in a car | Young | -0.62 | Middle | 0.00 | 0.63 | 0.003 |

Same abbreviations and conventions as before.

1. ***PROMIS Sleep-Related Impairment (PROMIS-SRI)***
2. *Rating scale functioning*

| **Score** | **Observed**  **count** | **%** | **Observed**  **average** | **Modal**  **thresholds** |
| --- | --- | --- | --- | --- |
| 1 | 846 | 17 | -1.27 | - |
| 2 | 1442 | 29 | -0.64 | -1.44 |
| 3 | 1495 | 30 | -0.10 | -0.44 |
| 4 | 813 | 16 | 0.38 | 0.68 |
| 5 | 428 | 9 | 0.58 | 1.20 |

Same abbreviations and conventions as above.

1. *Fit to the model – run 1*

|  |  |  | **Infit** | | **Outfit** | |
| --- | --- | --- | --- | --- | --- | --- |
| **Items** | **Calibration** | **SE** | **MNSQ** | **ZSTD** | **MNSQ** | **ZSTD** |
| 119 - alert | -0.85 | 0.06 | ***2.16 **** | 9.90 | ***2.61 **** | 9.90 |
| 120 - ready to start | -0.67 | 0.06 | ***1.87 **** | 9.52 | ***1.97 **** | 9.90 |
| 4 - energy | -0.53 | 0.06 | ***1.50 **** | 5.92 | ***1.59 **** | 6.81 |
| 19 - Sleep whenever | 0.25 | 0.06 | 1.17 | 2.13 | 1.17 | 2.08 |
| 123 - difficulty waking | 0.07 | 0.06 | 1.00 | 0.00 | 1.00 | 0.02 |
| 30 - irritable | 0.27 | 0.06 | 0.91 | -1.17 | 0.89 | -1.45 |
| 33 - emotions | 0.47 | 0.06 | 0.90 | -1.36 | 0.84 | -2.06 |
| 7 - awake | 0.34 | 0.06 | 0.84 | -2.14 | 0.82 | -2.45 |
| 6 - sleepy | -0.31 | 0.06 | 0.77 | -3.31 | 0.80 | -2.91 |
| 29 - daytime activities | 0.30 | 0.06 | 0.79 | -2.91 | 0.77 | -3.21 |
| 124 - sleepy woke up | -0.13 | 0.06 | 0.77 | -3.32 | 0.76 | -3.42 |
| 10 - getting things | 0.26 | 0.06 | 0.70 | -4.50 | 0.67 | -4.84 |
| 11 - concentrating on being sleepy | 0.11 | 0.06 | 0.67 | -4.90 | 0.69 | -4.53 |
| 25 - day problems | 0.31 | 0.06 | 0.68 | -4.80 | 0.66 | -5.04 |
| 27 - concentrating | 0.18 | 0.06 | 0.66 | -5.16 | 0.66 | -5.04 |
| 18 - tired | -0.07 | 0.06 | 0.60 | -6.22 | 0.61 | -6.07 |

Same abbreviations and conventions as above.

1. *Fit to the model – run 2*

The analysis was repeated after removing the most misfitting item, 119 – alert, i.e., the most misfitting one. Misfit of items 120 - ready to start, and 4 – energy was confirmed.

|  |  |  | **Infit** | | **Outfit** | |
| --- | --- | --- | --- | --- | --- | --- |
| **Items** | **Calibration** | **SE** | **MNSQ** | **ZSTD** | **MNSQ** | **ZSTD** |
| 120 - ready to start | -0.79 | 0.06 | ***2.27 **** | 9.90 | ***2.53 **** | 9.90 |
| 4 - energy | -0.65 | 0.06 | ***1.77 **** | 8.50 | ***1.95 **** | 9.90 |
| 19 - sleep whenever | 0.21 | 0.06 | 1.25 | 3.02 | 1.24 | 2.86 |
| … | … | … | … | … | … | … |

Same abbreviations and conventions as before. For conciseness, only misfitting items are reported, along with the first item that shows a proper fit.

1. *Fit to the model – run 3*

The analysis was repeated after simultaneously removing items 119 (alert) and 120 (ready to start). Misfit of item 4 - energy was confirmed.

|  |  |  | **Infit** | | **Outfit** | |
| --- | --- | --- | --- | --- | --- | --- |
| **Items** | **Calibration** | **SE** | **MNSQ** | **ZSTD** | **MNSQ** | **ZSTD** |
| 4 - energy | -0.78 | 0.07 | ***2.20 **** | 9.90 | ***2.61 **** | 9.90 |
| 19 - sleep whenever | 0.17 | 0.07 | 1.36 | 4.15 | 1.32 | 3.62 |
| … | … | … | … | … | … | … |

Same abbreviations and conventions as before. For conciseness, only misfitting items are reported, along with the first item that shows a proper fit.

1. *Fit to the model – run 4*

The analysis was repeated after removing items 119 (alert), 120 (ready to start), and 4 (energy). All remaining items subsequently demonstrated a good fit to the model.

|  |  |  | **Infit** | | **Outfit** | |
| --- | --- | --- | --- | --- | --- | --- |
| **Items** | **Calibration** | **SE** | **MNSQ** | **ZSTD** | **MNSQ** | **ZSTD** |
| 19 - sleep whenever | 0.12 | 0.07 | 1.49 | 5.42 | 1.46 | 5.02 |
| 123 - difficulty waking | -0.12 | 0.07 | 1.34 | 3.92 | 1.32 | 3.70 |
| 7 - awake | 0.25 | 0.07 | 1.12 | 1.44 | 1.08 | 1.00 |
| 30 - irritable | 0.15 | 0.07 | 1.10 | 1.21 | 1.05 | 0.59 |
| 33 - emotions | 0.43 | 0.08 | 1.10 | 1.22 | 1.01 | 0.17 |
| 6 - sleepy | -0.63 | 0.07 | 1.01 | 0.14 | 1.08 | 0.96 |
| 124 - sleepy woke up | -0.39 | 0.07 | 1.00 | 0.06 | 0.98 | -0.25 |
| 29 - daytime activities | 0.19 | 0.07 | 0.94 | -0.79 | 0.89 | -1.43 |
| 10 - getting things | 0.14 | 0.07 | 0.84 | -2.14 | 0.79 | -2.84 |
| 25 - day problems | 0.20 | 0.07 | 0.82 | -2.44 | 0.82 | -2.35 |
| 11 - concentrating on being sleepy | -0.06 | 0.07 | 0.79 | -2.81 | 0.79 | -2.75 |
| 18 - tired | -0.31 | 0.07 | 0.75 | -3.50 | 0.76 | -3.22 |
| 27 - concentrating | 0.03 | 0.07 | 0.74 | -3.63 | 0.72 | -3.81 |

Same abbreviations and conventions as above.

The rating scale analysis of the revised 13-item PROMIS SRI is reported in the following table.

| **Score** | **Observed**  **count** | **%** | **Observed**  **average** | **Modal**  **thresholds** |
| --- | --- | --- | --- | --- |
| 1 | 782 | 19 | -2.05 | - |
| 2 | 1287 | 32 | -1.05 | -2.14 |
| 3 | 1186 | 29 | -0.20 | -0.50 |
| 4 | 539 | 13 | 0.74 | 1.02 |
| 5 | 288 | 7 | 1.30 | 1.62 |

Same abbreviations and conventions as above.

1. *Differential Item Functioning*

No DIF was found for sex, age and physical activity.

1. ***Pittsburgh Sleep Quality Index***
2. *Rating scale functioning*

| **Item** | **Score** | **Observed**  **count** | **%** | **Observed**  **average** | **Modal**  **thresholds** |
| --- | --- | --- | --- | --- | --- |
| 1 | 0 | 94 | 30 | -1.31 |  |
|  | 1 | 111 | 35 | -0.83 | -1.04 |
|  | 2 | 83 | 26 | -0.45 | -0.12 |
|  | 3 | 26 | 8 | -0.03 | 1.16 |
| 2 | 0 | 65 | 21 | -1.45 |  |
|  | 1 | 111 | 35 | -1.02 | -1.03 |
|  | 2 | 81 | 6 | -0.51 | 0.30 |
|  | 3 | 57 | 8 | -0.08 | 0.73 |
| 3 | 0 | 127 | 40 | -1.20 | - |
|  | 1 | 111 | 35 | -0.87 | -0.69 |
|  | 2 | 38 | 12 | -0.20 | 0.69 |
|  | 3 | 38 | 12 | 0.12 | ***0.00**** |
| 4 | 0 | 178 | 57 | -1.13 |  |
|  | 1 | 45 | 14 | -0.69 | 0.66 |
|  | 2 | 27 | 9 | -0.52 | ***0.19 **** |
|  | 3 | 64 | 20 | -0.10 | ***-0.85 **** |
| 5 | 0 | 74 | 24 | -0.82 | - |
|  | 1 | 232 | 74 | -0.80 | -2.54 |
|  | 2 | 8 | 3 | -0.54 | 2.54 |
| 6 | 0 | 81 | 26 | -1.49 | - |
|  | 1 | 144 | 46 | -0.87 | -1.25 |
|  | 2 | 47 | 15 | -0.21 | 0.93 |
|  | 3 | 42 | 13 | 0.07 | ***0.32 **** |
| 7 | 0 | 186 | 59 | -1.03 | - |
|  | 1 | 101 | 32 | -0.52 | -1.60 |
|  | 2 | 26 | 8 | -0.34 | -0.37 |
|  | 3 | 1 | 0 | 1.31 | 1.97 |

Same abbreviations and conventions as above.

1. *Fit to the model*

|  |  |  | **Infit** | | **Outfit** | |
| --- | --- | --- | --- | --- | --- | --- |
| **Items** | **Calibration** | **SE** | **MNSQ** | **ZSTD** | **MNSQ** | **ZSTD** |
| 5 - disturbance | 0.40 | 0.13 | 1.22 | 2.41 | 1.29 | 2.80 |
| 4 - efficiency | -0.24 | 0.06 | 1.14 | 1.68 | 1.05 | 0.42 |
| 7 - daytime dysfunction | 1.41 | 0.09 | 1.06 | 0.72 | 1.10 | 1.05 |
| 1 - quality | -0.22 | 0.07 | 1.04 | 0.55 | 1.01 | 0.12 |
| 2 - latency | -0.73 | 0.07 | 0.92 | -1.12 | 0.89 | -1.52 |
| 3 - duration | -0.18 | 0.07 | 0.86 | -1.82 | 0.91 | -1.01 |
| 6 - meds | -0.45 | 0.07 | 0.79 | -3.01 | 0.75 | -3.42 |

Same abbreviations and conventions as above.

1. *Dimensionality*

The eigenvalue of the first principal component is 1.64, indicating unidimensionality.

1. *Differential Item Functioning*

| **Item** | **Group 1** | | **Group 2** | | **Contrast** | **p-value** |
| --- | --- | --- | --- | --- | --- | --- |
| 3 - duration | Males | -0.62 | Female | -0.07 | 0.55 | 0.002 |
| 3 - duration | Old | -0.86 | Young | 0.04 | 0.90 | <0.001 |

Same abbreviations and conventions as above.

**References**

1. Caronni A, Scarano S. *Generalisability of the Barthel Index and the Functional Independence Measure: robustness of disability measures to Differential Item Functioning.* Disabil Rehabil. 2025 Apr;47(8):2134-2145. doi: 10.1080/09638288.2024.2391554.

**Appendix**

Below are the score-to-measure tables for the four questionnaires.

1. ***Insomnia Severity Index***

| **Score** | **Measure** | **SE** |
| --- | --- | --- |
| 0 | -4.50 | 1.85 |
| 1 | -3.24 | 1.04 |
| 2 | -2.46 | 0.77 |
| 3 | -1.97 | 0.65 |
| 4 | -1.60 | 0.58 |
| 5 | -1.29 | 0.53 |
| 6 | -1.02 | 0.50 |
| 7 | -0.79 | 0.47 |
| 8 | -0.58 | 0.45 |
| 9 | -0.39 | 0.43 |
| 10 | -0.21 | 0.42 |
| 11 | -0.04 | 0.41 |
| 12 | 0.12 | 0.40 |
| 13 | 0.28 | 0.39 |
| 14 | 0.44 | 0.39 |
| 15 | 0.59 | 0.40 |
| 16 | 0.75 | 0.40 |
| 17 | 0.92 | 0.41 |
| 18 | 1.09 | 0.43 |
| 19 | 1.29 | 0.46 |
| 20 | 1.52 | 0.50 |
| 21 | 1.80 | 0.57 |
| 22 | 2.19 | 0.69 |
| 23 | 2.84 | 0.97 |
| 24 | 4.00 | 1.80 |

**The measure** and standard error of measurement (**SE**) are expressed in logit units. **Score:** questionnaire's total ordinal score.

1. ***Epworth Sleepiness Scale***

| **Score** | **Measure** | **SE** |
| --- | --- | --- |
| 0 | -5.28 | 1.86 |
| 1 | -3.99 | 1.06 |
| 2 | -3.18 | 0.79 |
| 3 | -2.65 | 0.68 |
| 4 | -2.23 | 0.61 |
| 5 | -1.88 | 0.57 |
| 6 | -1.57 | 0.55 |
| 7 | -1.28 | 0.53 |
| 8 | -1.01 | 0.51 |
| 9 | -0.76 | 0.50 |
| 10 | -0.51 | 0.50 |
| 11 | -0.26 | 0.49 |
| 12 | -0.02 | 0.49 |
| 13 | 0.23 | 0.50 |
| 14 | 0.48 | 0.50 |
| 15 | 0.73 | 0.51 |
| 16 | 0.99 | 0.52 |
| 17 | 1.27 | 0.53 |
| 18 | 1.56 | 0.55 |
| 19 | 1.88 | 0.58 |
| 20 | 2.24 | 0.62 |
| 21 | 2.67 | 0.69 |
| 22 | 3.21 | 0.80 |
| 23 | 4.05 | 1.07 |
| 24 | 5.35 | 1.86 |

Same abbreviations and conventions as above.

1. ***Revised (13 items) PROMIS Sleep-Related Impairment***

| **Score** | **Measure** | **SE** |
| --- | --- | --- |
| 13 | -6.53 | 1.84 |
| 14 | -5.28 | 1.03 |
| 15 | -4.52 | 0.75 |
| 16 | -4.05 | 0.63 |
| 17 | -3.69 | 0.57 |
| 18 | -3.40 | 0.52 |
| 19 | -3.14 | 0.49 |
| 20 | -2.91 | 0.47 |
| 21 | -2.70 | 0.45 |
| 22 | -2.50 | 0.44 |
| 23 | -2.31 | 0.43 |
| 24 | -2.13 | 0.42 |
| 25 | -1.96 | 0.41 |
| 26 | -1.79 | 0.41 |
| 27 | -1.62 | 0.40 |
| 28 | -1.46 | 0.40 |
| 29 | -1.30 | 0.40 |
| 30 | -1.15 | 0.39 |
| 31 | -0.99 | 0.39 |
| 32 | -0.84 | 0.39 |
| 33 | -0.69 | 0.39 |
| 34 | -0.54 | 0.38 |
| 35 | -0.39 | 0.38 |
| 36 | -0.25 | 0.38 |
| 37 | -0.11 | 0.38 |
| 38 | 0.03 | 0.37 |
| 39 | 0.17 | 0.37 |
| 40 | 0.31 | 0.37 |
| 41 | 0.44 | 0.36 |
| 42 | 0.57 | 0.36 |
| 43 | 0.70 | 0.36 |
| 44 | 0.83 | 0.35 |
| 45 | 0.95 | 0.35 |
| 46 | 1.08 | 0.35 |
| 47 | 1.20 | 0.35 |
| 48 | 1.32 | 0.35 |
| 49 | 1.45 | 0.35 |
| 50 | 1.57 | 0.35 |
| 51 | 1.69 | 0.35 |
| 52 | 1.82 | 0.36 |
| 53 | 1.95 | 0.36 |
| 54 | 2.08 | 0.37 |
| 55 | 2.22 | 0.38 |
| 56 | 2.37 | 0.39 |
| 57 | 2.53 | 0.40 |
| 58 | 2.70 | 0.42 |
| 59 | 2.89 | 0.45 |
| 60 | 3.11 | 0.48 |
| 61 | 3.36 | 0.53 |
| 62 | 3.68 | 0.6 |
| 63 | 4.11 | 0.73 |
| 64 | 4.83 | 1.01 |
| 65 | 6.05 | 1.83 |

Same abbreviations and conventions as above.

1. ***Pittsburgh Sleep Quality Index***

| **Score** | **Measure** | **SE** |
| --- | --- | --- |
| 0 | -4.48 | 1.87 |
| 1 | -3.17 | 1.07 |
| 2 | -2.34 | 0.79 |
| 3 | -1.82 | 0.66 |
| 4 | -1.44 | 0.58 |
| 5 | -1.13 | 0.52 |
| 6 | -0.88 | 0.48 |
| 7 | -0.66 | 0.45 |
| 8 | -0.47 | 0.43 |
| 9 | -0.29 | 0.42 |
| 10 | -0.11 | 0.42 |
| 11 | 0.06 | 0.42 |
| 12 | 0.25 | 0.44 |
| 13 | 0.45 | 0.46 |
| 14 | 0.68 | 0.50 |
| 15 | 0.96 | 0.55 |
| 16 | 1.31 | 0.64 |
| 17 | 1.79 | 0.76 |
| 18 | 2.50 | 0.93 |
| 19 | 3.60 | 1.19 |
| 20 | 5.08 | 1.93 |

Same abbreviations and conventions as above.
